# Efp/TRIM25 and Its Related Protein, TRIM47, in Hormone-Dependent Cancers

**DOI:** 10.3390/cells11152464

**Published:** 2022-08-08

**Authors:** Kotaro Azuma, Satoshi Inoue

**Affiliations:** 1 Department of Geriatric Medicine, Graduate School of Medicine, The University of Tokyo, Tokyo 113-8655, Japan; 2 Department of Systems Aging Science and Medicine, Tokyo Metropolitan Institute of Gerontology, Tokyo 173-0015, Japan; 3 Division of Systems Medicine and Gene Therapy, Saitama Medical University, Saitama 350-1241, Japan

**Keywords:** estrogen-responsive finger protein (Efp), TRIM family proteins, TRIM25, TRIM47, hormone-dependent cancers, breast cancer, endometrial cancer, prostate cancer

## Abstract

Increasing attention has been paid to the biological roles of tripartite motif-containing (TRIM) family proteins, which typically function as E3 ubiquitin ligases. Estrogen-responsive finger protein (Efp), a member of the TRIM family proteins, also known as TRIM25, was originally identified as a protein induced by estrogen and plays critical roles in promoting endocrine-related cancers, including breast cancer, endometrial cancer, and prostate cancer. The pathophysiological importance of Efp made us interested in the roles of other TRIM family proteins that share a similar structure with Efp. Based on a phylogenetic analysis of the C-terminal region of TRIM family proteins, we focused on TRIM47 as a protein belonging to the same branch as Efp. TRIM47 is a poor prognostic factor in both breast cancer and prostate cancer. Atypical lysine-27-like poly-ubiquitination was involved in the underlying mechanism causing endocrine resistance in breast cancer. We also discuss the functions of Efp and TRIM47 in other types of cancers and innate immunity by introducing substrates the are modified by poly-ubiquitination.

## 1. Introduction

We previously identified a human, estrogen-induced protein named Efp (estrogen-responsive finger protein) [1]. The protein was later classified as TRIM25, a member of the tripartite motif-containing (TRIM) family of proteins, which typically have a common structure containing RING finger, B-box, and coiled-coil domains and consist of more than 70 members sharing the domain structure [2]. We demonstrated that the functions of Efp contributed to breast cancer progression [3], which led to our interest in the roles of other Efp-related TRIM family proteins. From phylogenetic analysis of C-terminal domains of TRIM family [4], several proteins are shown to have a close relationship with Efp. Among them, we focused on TRIM47, which was recently shown to be related to endocrine resistance in breast cancer [5]. In this review article, we introduce functions of Efp and TRIM47, including their roles in endocrine-related cancers.

## 2. Efp and Breast Cancer

Breast cancer is the most prevalent malignancy in women worldwide [6]. Among breast cancer cases, approximately 70% of them are tumors expressing estrogen receptor alpha (ERα) and diagnosed as estrogen receptor (ER)-positive breast cancer [7]. For treatment of ER-positive breast cancer, suppression of ER function is important as well as surgical resection, indicating that ER signaling promotes development of breast cancer. Several kinds of endocrine therapies including aromatase inhibitors, gonadotropin-releasing hormone analogues, selective estrogen receptor modulators, and selective estrogen receptor degraders are used for endocrine therapy for breast cancer [7].

ER signaling is classified into two functions: genomic action and non-genomic action [8]. In genomic action, ER functions as a ligand-dependent transcription factor by binding with the estrogen-responsive element (ERE) that exists in the enhancer region of target genes upon the stimulation of estrogen. Then, ER forms a complex with coactivators having histone acetyltransferase activity, resulting in conformational changes of chromatin and activation of transcription pathways [9,10,11]. Estrogen-dependent proteins including Efp [3], Cyclin D1 [12], and GREB1 [13] are reported to be associated with development of breast cancer. Estrogen-induced long non-coding RNAs such as *HOTAIR* are also involved in breast cancer progression [14]. In non-genomic action, ER interacts with cytoplasmic protein ligands dependently and causes a rapid change of cellular function. On stimulation with estrogen, rapid phosphorylation of MAPK mediated by ER was observed in breast cancer cells [15]. We previously reported rapid deacetylation of tubulin on estrogen treatment that is mediated by complex formation of ER, HDAC6, and tubulin in the proximity of the plasma membrane [16]. These two kinds of ER signaling, namely genomic and non-genomic signaling, provide a mechanistic background for breast cancer treatment targeting ER.

**Figure 1:**
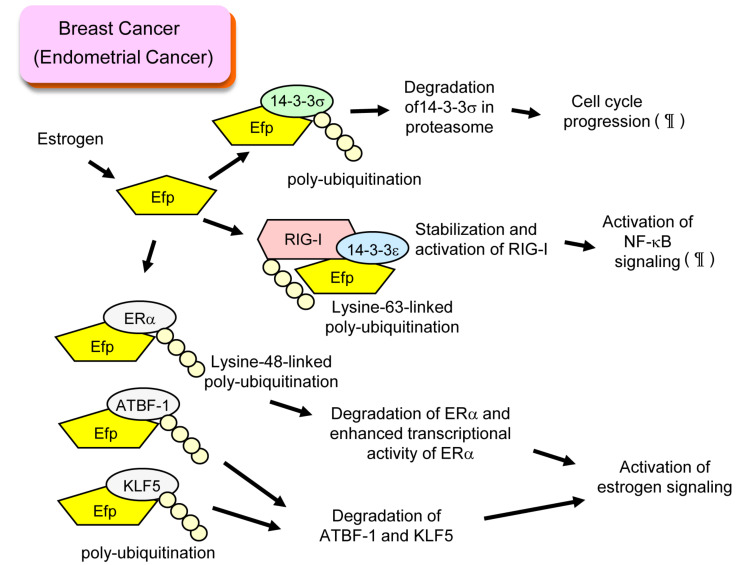
Roles of Efp in breast cancer and endometrial cancer. In breast cancer cells, Efp is transcriptionally induced by estrogen and contributes to cancer progression with several mechanisms. ¶: Mechanisms also suggested in endometrial cancer.

Efp is a representative estrogen-induced protein that contributes to breast cancer progression. The *EFP* gene was one of the genes we discovered by isolating genomic fragments associating with the DNA-binding domain of ER [17]. Since the Efp protein has a RING finger domain that is a characteristic of a protein with ubiquitin ligase activity, we assumed the binding partners of Efp as candidates of ubiquitinated substrates. Finally, we identified 14-3-3σ, a cell cycle checkpoint protein that inhibits G2/M progression, as a substrate of Efp [3]. Efp-dependent poly-ubiquitination of 14-3-3σ led to proteasomal degradation of 14-3-3σ and proliferation of breast cancer cells (Figure 1). Typically, the ubiquitin code used for proteasomal degradation is lysine-48-linked poly-ubiquitination [18]. However, the precise analysis of the Efp-dependent poly-ubiquitination of the 14-3-3σ protein has not been reported. We also demonstrated that breast cancer patients with stronger Efp immunoreactivity displayed a significantly poor prognosis [19]. By using mouse xenografts, we demonstrated that intra-tumor injection of siRNA targeting Efp suppressed the growth of xenograft tumor, suggesting that Efp could be a therapeutic target [20].

In non-malignant tissues, Efp is also known to have physiological roles related to innate immunity, as explained later. We previously demonstrated that retinoic acid-inducible gene I (RIG-I, also known as DEAD box polypeptide 58; DDX58) was modified by lysine-63-linked poly-ubiquitination by Efp [21]. This led to activation of RIG-I, which is shown by interferon-β production, activation of NF-κB signaling, and antiviral activity in response to RNA virus infection. Interestingly, a member of the 14-3-3 proteins, 14-3-3ε, is involved in the formation of complexes including Efp and RIG-I [22]. When we examine the immunoreactivity of RIG-I in ER-positive breast cancer tissues, stronger immunoreactivity of RIG-I was related to significantly poorer prognosis of breast cancer patients [23]. We also discovered the positive correlation of RIG-I and Efp immunoreactivity [23], which may suggest that RIG-I is stabilized and activated by Efp also in breast cancer cells and is involved in breast cancer progression (Figure 1).

Interestingly, ERα was reported to be a substrate of Efp in ER-positive breast cancer cells [24]. Efp-dependent, lysine-48-linked poly-ubiquitination of ERα was shown. This poly-ubiquitination promoted degradation of ERα, whereas transcriptional activity of ERα was rather enhanced in the presence of Efp. Efp lacking RING finger domains suppressed transcriptional activity of ERα, suggesting the importance of E3 ubiquitin activity in ERα transcriptional activity (Figure 1).

Two other transcription factors, Krüppel-like factor 5 (KLF5) and AT motif binding factor 1 (ATBF-1), are also reported as substrates of Efp in ER-positive breast cancer [25,26]. The estrogen-dependent degradation of these proteins was observed, which was explained by concomitant induction of Efp and poly-ubiquitination of these proteins. Since both KLF5 and ATBF-1 were shown to suppress estrogen signaling in breast cancer cells [27,28], Efp is suggested to promote breast cancer by enhancing estrogen receptor signaling (Figure 1).

## 3. Efp and Endometrial Cancer

Similar functions of Efp are supposed to be underlying mechanisms of endometrial cancer development. Most endometrial cancers are considered to be estrogen-related because they develop in response to prolonged and unopposed estrogen stimulation. We showed that the amount of 14-3-3σ protein increased by knocking down Efp expression, which was accompanied by suppression of cell cycle [29]. This result suggested the estrogen-dependent induction of Efp may promote endometrial cancer growth by degrading 14-3-3σ protein as in breast cancer cells (Figure 1). We also showed Efp-dependent activation of NF-κB signaling [29], which suggested a RIG-I-mediated mechanism might also exist in endometrial cancer (Figure 1).

Recently, we succeeded in three-dimensional culture of endometrial-cancer-patient-derived cancer cells (EC-PDC) which express high amounts of ERα [30]. This model provides an authentic model of endometrial cancer, recapitulating clinical pathophysiology. Interestingly, estradiol, which often has a suppressive role in inflammation, up-regulated inflammation-associated genes in EC-PDC. We also observed induction of *EFP* by estrogen treatment of EC-PDC [31]. Knockdown of *EFP* by siRNA resulted in suppressed spheroid formation of EC-PDC, which further supported the importance Efp in promotion of clinical endometrial cancer. Knockdown of *EFP* suppressed inflammation-related genes as well, which suggested that the inflammation promoting effect of estrogen in endometrial cancer cells could be mediated by Efp induction.

## 4. Efp and Prostate Cancer

**Figure 2:**
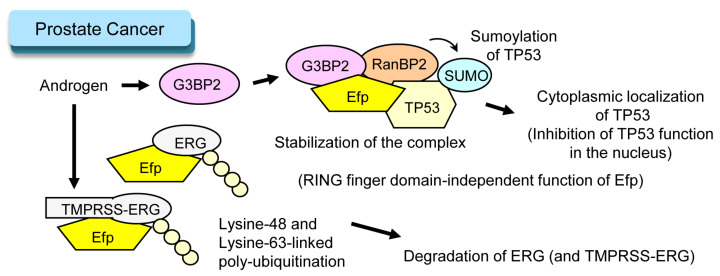
Roles of Efp in prostate cancer. Efp is involved in function of an androgen-dependent protein, G3BP2. This mechanism is independent of E3 ubiquitin ligase activity of Efp. Efp was also reported to be involved in degradation of a fusion protein encoded by *TMPRSS2-ERG* fusion gene, which seems to be tumor suppressive. Note that transcription of *TMPRSS2/ERG* is induced by androgen.

Prostate cancer is another example of endocrine-related cancer. For treatment of prostate cancer suppression of androgen receptor (AR) function is important. Several kinds of endocrine therapies including castration, gonadotropin-releasing hormone analogues and AR antagonists are used as androgen deprivation therapy (ADT) for prostate cancer [32]. Interestingly, Efp is involved in androgen-dependent prostate cancer progression in a RING-finger-domain-independent manner. Androgen-induced GTPase-activating protein-binding protein 2 (G3BP2), which causes TP53 translocation from the nucleus to cytoplasm by forming a complex with Ran binding protein 2 (RanBP2), which is a SUMO E3 ligase and is responsible for sumoylation of TP53 [33]. Efp stabilizes the complex including TP53, G3BP2, and RanBP2, which inhibits the tumor suppressive function of TP53 in the nucleus [34] (Figure 2). Indeed, stronger immunoreactivity of Efp was a poor prognostic factor for prostate cancer patients. The tumor promoting effect of Efp remained when the RING finger domain was deleted, which suggested that the function of Efp in prostate cancer does not require E3 ubiquitin ligase activity [34].

ERG is a protein encoded by a gene called *E-twenty-six (Ets)-related gene (ERG)*. In prostate cancer, *ERG* often forms a fusion gene with *transmembrane protease, serine 2 (TMPRSS2)* [35]. Since TMPRSS2 is an androgen-dependent gene, the fusion protein encoded by TMPRSS2/ERG is overexpressed in prostate cancer, which contributes to tumor progression [35]. Efp was shown to interact with the ERG protein and caused RING-finger-domain-dependent poly-ubiquitination of the ERG protein, which led to degradation of the ERG protein [36]. It was also shown that Efp was able to interact with ERG protein without an N-terminus region, lacking in the protein encoded by TMPRSS2/ERG fusion gene. Considering the pathological role of the TMPRSS2/ERG fusion gene, this function of Efp could seem to be tumor suppressive (Figure 2).

## 5. Roles of Efp in Other Cancers

Recently, the biological roles of Efp have begun to be elucidated in several cancer types other than hormone-dependent cancers. In this process, new substrates for Efp as an E3 ubiquitin ligase have been discovered.

In hepatocellular carcinoma (HCC), metastasis-associated 1 (MTA-1), which is involved in metastatic progression, was shown to be a substrate of Efp [37]. Poly-ubiquitination and degradation of MTA-1 by Efp was shown. Efp suppressed migration of the HCC cell line, HuH6, suggesting that Efp has tumor suppressive roles in HCC. On the other hand, Efp seems to enhance proliferation of HCC cells. It was shown that Efp increased lysine-48-linked poly-ubiquitination of F-box and WD repeat domain-containing 7α (FBXW7α), which is another E3 ubiquitin ligase responsible for degradation of the Myc protein. Efp induced degradation of FBXW7α followed by stabilization of the Myc protein [38]. Another mechanism was reported for promoting proliferation of HCC. Efp was reported to be responsible for poly-ubiquitination and degradation of Keap1, which is involved in the endoplasmic reticulum stress response [39]. Degradation of Keap1 by Efp facilitated nuclear translocation of Nrf2, which contributed tumor cell survival under endoplasmic reticulum stress.

In gastric cancer, Efp was found to cause poly-ubiquitination of specific protein 1 (SP1), a transcription factor that induces transcription of proteins including matrix metalloproteinases 2 (MMP2) and is involved in tumor growth, metastasis, and angiogenesis. Efp caused degradation of SP1, and high expression of Efp was related to the favorable prognosis of gastric cancer patients [40]. In thyroid cancer, Efp was shown to cause poly-ubiquitination of DEAD-box protein 5 (DDX5) [41]. DDX5 is a member of the DEAD-box polypeptides, which also includes RIG-I (also known as DEAD-box polypeptide 58). DDX5 interacts with the transcription factor E2F1 and promotes thyroid cancer development. Therefore, Efp may have a tumor suppressive function in thyroid cancer. It is of note that interaction of Efp and DDX5 requires SLC26A4-AS, suggesting the feature of Efp as an RNA-binding protein. In a study mainly using glioma cells, Efp was reported to be responsible for poly-ubiquitination and degradation of Capicua, a protein functioning as a transcriptional repressor of receptor tyrosine kinase signaling [42]. In this context, Efp has a tumor promotive effect. It is noteworthy that Capicua interacts with 14-3-3 proteins, although interaction with 14-3-3σ was not evaluated [43]. In non-small cell lung cancer, Efp was shown to modify phosphatase and tensine homolog (PTEN) with lysine-63-linked poly-ubiquitination [44]. PTEN is a phosphatase that negatively regulates the PI3K/Akt pathway. Lysine-63-linked poly-ubiquitination of PTEN prevented its translocation to the plasma membrane, which disturbs its function as a phosphatase. Therefore, Efp results in activation of PI3K/Akt signaling, and contributes to tumor promotion.

So far, we have explained the functions of Efp as an E3 ubiquitin ligase in several cancers. The known substrates of Efp are summarized in Table 1. Intriguingly, Efp has different modes of action other than ubiquitination of substrate proteins. One of the mechanisms is its function as an RNA-binding protein. Interaction of Efp with RNAs plays important roles in the regulation of innate immunity introduced below, whereas in cancer biology, its protective role is shown in colon cancer cells. Efp was shown to bind caspase-2 mRNA and to prevent translation of caspase-2 [45]. This function of Efp was related to decreased apoptosis in response to chemotherapeutic drugs. Another mode of Efp action is a function as a transcription modulator. In HCC, Efp suppressed expression of TRIM19, a tumor suppressive protein also known as promyelocytic leukemia protein (PML). In this study, association of Efp at the promoter region of *TRIM19* was shown by chromatin immunoprecipitation [46], suggesting Efp functions as a transcription modulator.

## 6. Roles of Efp in Innate Immunity

Although the function of Efp was originally studied in the context of estrogen-dependent cancer, its physiological roles in innate immunity have attracted attention. Moreover, some of the processes seem to be shared with cancer biology, as explained earlier in relation to RIG-I protein. Therefore, we consider that it is worthwhile summarizing the roles of Efp in innate immunity in this review article.

The relationship of Efp with innate immunity was originally recognized by our findings that Efp was responsible for the lysine-63-linked poly-ubiquitination of RIG-I [21]. In the process of innate immunity, antigen-independent recognition of pathogens is important, and is performed by molecules called pattern recognition receptors (PRRs) including RIG-I and melanoma differentiation-associated gene 5 (MDA5). RIG-I has high affinity with short dsRNAs with tri-phosphorylation at their 5′ ends, whereas MDA5 is preferentially associated with long dsRNAs [49]. When RIG-I or MDA5 binds with a dsRNA, it forms a helical oligomer that activates mitochondrial antiviral signaling proteins (MAVS), also known as VISA (virus-induced signaling adaptor), IPS-1 (IFN beta-promoter stimulator 1), or Cardif (CARD adapter-inducing interferon beta). Activation of MAVS stimulates NF-κB signaling or induces type-1 interferon genes [49]. We previously showed that Efp is responsible for the lysine-63-linked poly-ubiquitination of two caspase recruitment domains (2CARD) in the N-terminal region of RIG-I, which is required to activate RIG-I. Murine embryonic fibroblasts (MEFs) derived from Efp knockout mice displayed decreased production of Sendai-virus-induced IFN-β and increased infection with vesicular stomatitis virus (VSV) [21]. Recently, other E3 ubiquitin ligases, namely RING finger protein leading to RIG-I activation (Riplet) [50], mex-3 RNA-binding family member C (MEX3C) [51], and TRIM4 [52], are shown to be responsible for the poly-ubiquitination and activation of RIG-I. It was proposed that Riplet has a vital role for activating RIG-I by catalyzing poly-ubiquitination of the C-terminal region of RIG-I, then Efp, MEX3C, and TRIM4 become able to add poly-ubiquitin chains at 2CARD in the N-terminal region of RIG-I [53].

Efp is also involved in the regulation of innate immunity by activating a protein called zinc-finger antiviral protein (ZAP). ZAP binds to viral RNA directly or binds to viral mRNA, which induces their degradation. Efp was reported to be responsible for lysine-48- and lysine-63-linked poly-ubiquitination of ZAP [47,48]. Lysine-63-linked poly-ubiquitination was shown to be important for the antiviral activity of ZAP.

So far, the function Efp as an E3 ubiquitin ligase on activating two RNA binding proteins, RIG-I and ZAP, has been described. Interestingly, Efp itself is an RNA-binding protein, and the function described above is even dependent on its characteristic as an RNA binding protein. It was shown that Efp binds to a long noncoding RNA, Lnczc3h7a, which also associated with RIG-I [54]. Thus, Lnczc3h7a serves as a scaffold to stabilize the Efp–RIG-I complex. Moreover, the RNA binding characteristic of Efp is supposed to also be important for activating ZAP by analysis using a deletion mutant of Efp lacking residues 470–508 in the PRY/SPRY domain that are responsible for RNA binding [55].

Finally, we would like to mention the relationship of Efp with SARS-CoV-2. SARS-CoV-2 causes coronavirus disease 2019 (COVID-19) which has spread worldwide. One of the proteins produced by SARS-CoV-2, called nucleocapsid, was shown to form complexes with Efp. This interaction impaired Efp activity as an E3 ubiquitin ligase, and suppressed activation of RIG-I [56]. Thus, Efp can be one of the targets that SARS-Cov-2 utilizes to escape from innate immunity.

## 7. TRIM47 and Cancers

**Figure 3:**
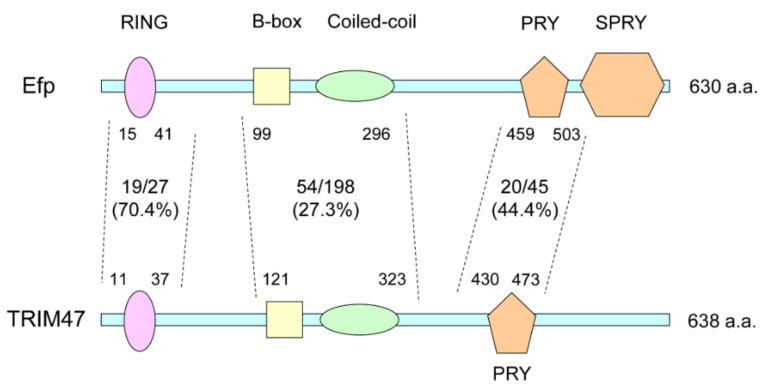
Homology of human TRIM47 with Efp. The number and ratio of identical amino acids in RING finger domain (RING), B-box and coiled-coil domains, and PRY domain are shown. The numbers of amino acids are based on that of Efp when the ratios are calculated. a.a.: amino acids.

The functions of Efp and their pathophysiological importance led our interest to the roles of other TRIM family proteins that share a similar structure with Efp. According to a phylogenetic analysis of the PRY/SPRY domain that often exists in the C-terminal region of TRIM family proteins, the Efp protein has similar structure to TRIM65, RIPLET, TRIM16, TRIM16L, and TRIM47 [4]. Among these, we focused on TRIM47, which was recently shown to be related to endocrine resistance in breast cancer [5]. The numbers of identical amino acids to Efp in each domain of TRIM47 are indicated in Figure 3. Based on the homology search using BLAST (basic local alignment search tool) provided by NCBI (National Center for Biotechnology Information; https://www.ncbi.nlm.nih.gov/ accessed on 12 January 2022), TRIM47 shares identical amino acids with Efp in 19 out of 27 amino acids (70.4%) in the region including the RING finger domain, 54 out of 198 (27.3%) in the region including B-box and coiled-coil domains, and 20 out of 45 (44.4%) in the region including PRY domain (Figure 3).

*TRIM47* was originally identified as an overexpressed gene in astrocytoma [57]. It is also shown to be a gene associated with leukoaraiosis [58] together with TRIM65, which is also shown to be phylogenetically close to Efp [4]. TRIM47 is also known to contribute to pathogenesis of nonalcoholic steatohepatitis (NASH) by causing ubiquitination and degradation of a protein called cylindromatosis (CYLD) [59]. In terms of inflammation, TRIM47 was reported to be involved in acute lung injury caused by lipopolysaccharide. As a mechanism for acute lung injury, inflammation of endothelial cells was caused by activation of TRAF2 with lysine-63-linked polyubiquitination mediated by TRIM47 [60].

In the relationship with cancers, TRIM47 was shown to be responsible for ubiquitination and degradation of SMAD4, which led to chemoresistance in response to 5-fluorouracil (5-FU) therapy in colorectal cancer [61]. In glioma cells, TRIM47 enhanced cell proliferation and migration by ubiquitination and degradation of FOXO1 [62]. In pancreatic cancer, TRIM47 causes ubiquitination and degradation of fructose-1, 6-biphosphatase 1 (FBP1) [63]. FBP1 is a key enzyme in gluconeogenesis and catalyzes the opposite reaction to phosphofructokinase-1 (PFK1), which is an important enzyme in glycolysis. Thus, degradation of FBP1 enhances the Warburg effect by promoting glycolysis, and contributes to the progression of pancreatic cancer [63]. In renal cell carcinoma, TRIM47 promoted proliferation with ubiquitination and degradation of TP53 [64].

## 8. TRIM47 and Hormone-Dependent Cancers

**Figure 4:**
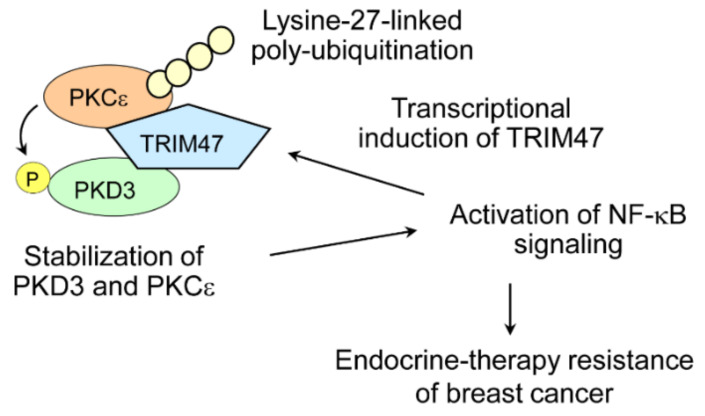
A role of TRIM47 in endocrine-resistant breast cancer. TRIM47 binds with PKCε and PKD3. PKCε is modified with lysine-27-linked poly-ubiquitination in a TRIM47-dependent manner. PKD3 is phosphorylated by PKCε. Here, TRIM47 serves as a E3 ubiquitin ligase for PKCε and a scaffold to form a ternary complex of TRIM47/PKCε/PKD3. The formation of this ternary complex caused stabilization of PKCε and PKD3, which led to activation of NF-κB signaling. Activation of NF-κB signaling is a known to promote endocrine resistance in breast cancer. NF-κB signaling also stimulates transcription of TRIM47, which forms feed forward loop.

In terms of hormone-dependent cancers, we previously showed that TRIM47 is a poor prognostic factor for prostate cancer patients [65]. As for breast cancer, two independent studies including ours showed tumor promoting effects of TRIM47 [5,66]. We demonstrated that overexpression of the TRIM47 protein predicted poor prognosis in ER-positive breast cancer patients, and that overexpression of TRIM47 caused endocrine-therapy-resistant growth in breast cancer cell lines [5]. In contrast to Efp, TRIM47 is not induced by estrogen. In fact, expression of TRIM47 was induced by NF-κB signaling. On the other hand, we showed TRIM47 causes activation of NF-κB signaling, forming a positive feedback loop (Figure 4). Our results were in line with previous reports that showed that enhanced NF-κB signaling is one of the causes of endocrine resistance in breast cancer [67,68]. Mechanistically, TRIM47 was associated with two kinases, namely protein kinase C epsilon (PKCε) and protein kinase D3 (PKD3), both of which are known to activate NF-κB signaling [69,70]. TRIM47 increased the stability of these two kinases and this effect was RING finger domain dependent. We revealed TRIM47-dependent PKCε ubiquitination involves atypical lysine-27-linked poly-ubiquitination [5]. In this model, stabilization and activation of PKD3 can be explained by phosphorylation of PKD3, since PKD3 was reported to be phosphorylated by PKCε in prostate cancer cells [71]. Thus, TRIM47 serves as a scaffold to bring PKD3 in proximity to PKCε (Figure 4).

## 9. TRIM47 and Innate Immunity

In contrast to Efp, the reports on the relationship between TRIM47 and innate immunity are limited. Recently, it was reported that TRIM47 was utilized by vesicular stomatitis virus (VSV) to evade innate immunity. VSV infection induced an immune checkpoint inhibitor protein called Tim-3 in macrophages. Tim3 interacted with TRIM47 and its substrate NF90 and facilitated TRIM47-dependent lysine-48-linked poly-ubiquitination of NF90. NF90 is a viral sensor that recognizes virus dsRNA and triggers innate immunity by formation of stress granules (SGs), an inhibitory machinery for viral replication. Interaction of Tim-3, TRIM47, and NF90 resulted in degradation of NF90, which promoted VSV replication [72]. All the known substrates of TRIM47, including NF90, are summarized in Table 2.

## 10. Conclusions

Almost 30 years have passed since we discovered Efp as an estrogen-induced protein. Since then, the functions of Efp were studied by many researchers. At first, the research area was mainly in the field of hormone-dependent cancers. Recently, the research on Efp has developed into other types of cancers, and innate immunity. Intriguingly, a molecule that has a physiological role in innate immunity such as RIG-I was found to have roles in cancer biology. Therefore, it is possible that other molecules in innate immunity could elucidate cancer biology, and vice versa. Here, structurally similar TRIM47 can also be included in these research areas. Based on the knowledge introduced in this review, one of the strategies to develop new drugs for the treatment of hormone-related cancers is to find small molecules that interfere with the associations of Efp or TRIM47 with their interacting molecules. However, as stated above, the same interactions, such as Efp and RIG-I, are sometimes involved in innate immunity. Careful attention should be paid to the side effects related to innate immunity when developing anti-tumor drugs targeting these molecules. Integrating the knowledge from oncology and innate immunity would be an attractive task, and it could contribute to the future works in these fields.

## Figures and Tables

**Table 1:** Substrates of Efp.

Substrate Proteins	Poly-Ubiquitination Code	Cancer	Function	References
14-3-3σ	unknown	Breast Cancer	degradation	[3]
RIG-I	Lys-63-linked	Breast Cancer ^1^	activation	[21,23]
ERα	Lys-48-linked	Breast Cancer	degradation/ activation	[24]
KLF5	unknown	Breast Cancer	degradation	[25]
ATBF-1	unknown	Breast Cancer	degradation	[26]
ERG	Lys-48, Lys-63-linked	Prostate Cancer	degradation	[36]
MTA-1	unknown	Hepatocellular carcinoma	degradation	[37]
FBXW7α	Lys-48-linked	Hepatocellular carcinoma	degradation	[38]
Keap1	unknown	Hepatocellular carcinoma	degradation	[39]
Sp1	unknown	Gastric cancer	degradation	[40]
DDX5	unknown	Thyroid cancer	degradation	[41]
Capicua	unknown	Brain tumors	degradation	[42]
PTEN	Lys-63-linked	Non-small cell Lung carcinoma	prevention of translocation	[44]
ZAP	Lys-48, Lys-63-linked	N/A ^2^	enhancing activity ^3^	[47,48]

^1^ This function is mainly related to innate immunity. ^2^ This function is related to innate immunity. ^3^ Lysine-63-linked poly-ubiquitination is responsible for this function. Lys: Lysine.

**Table 2:** Substrates of TRIM47.

Substrate Proteins	Poly-Ubiquitination Code	Cancer	Function	References
CYLD	unknown	N/A ^1^	degradation	[59]
TRAF2	Lys-63-linked	N/A ^2^	activation	[60]
SMAD4	unknown	Colorectal cancer	degradation	[61]
FOXO1	unknown	Glioma	degradation	[62]
FBP1	unknown	Pancreatic cancer	degradation	[63]
TP53	unknown	Renal cell carcinoma	degradation	[64]
PKCε	Lys-27-linked	Breast cancer	stabilization	[5]
NF90	Lys-48-linked	N/A ^3^	degradation	[72]

^1^ This function is related to nonalcoholic steatohepatitis. ^2^ This function is related to endothelial inflammation. ^3^ This function is related to innate immunity.

## Data Availability

Not applicable.
